# Hospital Effluents Are One of Several Sources of Metal, Antibiotic Resistance Genes, and Bacterial Markers Disseminated in Sub-Saharan Urban Rivers

**DOI:** 10.3389/fmicb.2016.01128

**Published:** 2016-07-22

**Authors:** Amandine Laffite, Pitchouna I. Kilunga, John M. Kayembe, Naresh Devarajan, Crispin K. Mulaji, Gregory Giuliani, Vera I. Slaveykova, John Poté

**Affiliations:** ^1^Faculty of Science, Earth and Environmental Science Section, F.-A. Forel Institute and Institute of Environmental Sciences, University of GenevaGeneva, Switzerland; ^2^Department of Chemistry, Faculty of Science, University of KinshasaKinshasa, Democratic Republic of the Congo; ^3^Département de Géographie-Science de l'Environnement, Faculté des Sciences, Université Pédagogique NationaleKinshasa, Democratic Republic of the Congo; ^4^enviroSPACE Lab., Institute for Environmental Sciences, University of GenevaGeneva, Switzerland; ^5^United Nations Environment Programme, Division of Early Warning and Assessment, Global Resource Information Database – Geneva, International Environment HouseGeneva, Switzerland

**Keywords:** hospital and urban wastewater, water pollution, sediment receiving system, toxic metals, antibiotic resistance genes, fecal indicator bacteria, Sub-Saharan Africa, tropical conditions

## Abstract

Data concerning the occurrence of emerging biological contaminants such as antibiotic resistance genes (ARGs) and fecal indicator bacteria (FIB) in aquatic environments in Sub-Saharan African countries is limited. On the other hand, antibiotic resistance remains a worldwide problem which may pose serious potential risks to human and animal health. Consequently, there is a growing number of reports concerning the prevalence and dissemination of these contaminants into various environmental compartments. Sediments provide the opportunity to reconstruct the pollution history and evaluate impacts so this study investigates the abundance and distribution of toxic metals, FIB, and ARGs released from hospital effluent wastewaters and their presence in river sediments receiving systems. ARGs (*bla*_TEM_, *bla*_CTX-M_, *bla*_SHV_, and *aadA*), total bacterial load, and selected bacterial species FIB [*Escherichia coli, Enterococcus* (ENT)] and species (Psd) were quantified by targeting species specific genes using quantitative PCR (qPCR) in total DNA extracted from the sediments recovered from 4 hospital outlet pipes (HOP) and their river receiving systems in the City of Kinshasa in the Democratic Republic of the Congo. The results highlight the great concentration of toxic metals in HOP, reaching the values (in mg kg^−1^) of 47.9 (Cr), 213.6 (Cu), 1434.4 (Zn), 2.6 (Cd), 281.5 (Pb), and 13.6 (Hg). The results also highlight the highest (*P* < 0.05) values of 16S rRNA, FIB, and ARGs copy numbers in all sampling sites including upstream (control site), discharge point, and downstream of receiving rivers, indicating that the hospital effluent water is not an exclusive source of the biological contaminants entering the urban rivers. Significant correlation were observed between (i) all analyzed ARGs and total bacterial load (16S rRNA) 0.51 to 0.72 (*p* < 0.001, *n* = 65); (ii) ARGs (except *bla*_TEM_) and FIB and Psd 0.57 < *r* < 0.82 (*p* < 0.001, *n* = 65); and (iii) ARGs (except *bla*_TEM_) and toxic metals (Cd, Cr, Cu, and Zn) 0.44 to 0.72, (*p* < 0.001, *n* = 65). These findings demonstrate that several sources including hospital and urban wastewaters contribute to the spread of toxic metals and biological emerging contaminants in aquatic ecosystems.

## Introduction

Contamination of freshwater resources with anthropogenic pollutants is a growing concern of interest because safe and readily available water is needed for drinking, domestic use, food production, and recreational purposes (WHO, 2015). Freshwater resource pollution by various contaminants including toxic metals, persistent organic pollutants, pathogenic organisms, antibiotic resistant bacteria (ARB), and antibiotic resistant genes (ARGs) is still a major problem in many parts of the world (Poté et al., 2008; Knapp et al., 2012; Bréchet et al., 2014; Czekalski et al., 2014; Devarajan et al., 2015b). The situation is particularly alarming in developing regions such as in Sub-Saharan Africa where most rivers, lakes, and lagoons are receiving untreated hospital and industrial effluent water, mining effluents, and urban storm water runoff affected by anthropogenic pollutants due to intensive and uncontrolled urbanization (Feng et al., 2004; Chatterjee et al., 2007; Gnandi et al., 2011; Atibu et al., 2013; Mwanamoki et al., 2014, 2015).

Hospital effluents are a particular case of anthropogenic pollutants. Indeed, hospital wastewaters are complex mixtures of chemical and biological substances which are continually discharged (Barcelo and Barceló, 2003; Boillot et al., 2008; Verlicchi et al., 2010). This mixture is the result of diagnostic laboratory and research activity waste and medicine excretion which include active principles from medicinal products and their metabolites, chemicals, disinfecting agents, specific detergents, radioactive markers, iodinated contrast media, nutrients, and bacteria and their antimicrobial resistance genes (Verlicchi et al., 2010). Particularly studied among these hospital contaminants are bacteria and their antimicrobial resistance genes because of their great ability to disseminate and their clinical and financial impact (Davies et al., 1995; Cosgrove, 2006). The remarkable effectiveness of antibiotics has reduced mortality linked to bacterial diseases in only a few decades but their over- and mis-use has rapidly lead to a dramatic exponential increase in antibiotic resistance and multidrug-resistant bacteria throughout the world (Davies and Davies, 2010; Fair and Tor, 2014). The speed with which resistance has spread is explained by acquired resistance. In contrast to chromosomal resistance which is responsible for resistance to an antibiotic or an antibiotic class, acquired resistance by genetic material acquisition may be responsible for resistance against many antibiotics or antibiotic classes (INSERM, 2013). This resistance is harbored by many human and animal, pathogenic, and potentially pathogenic bacteria and can easily be spread by either conjugation, transformation, or transduction of resistance genes which are generally located on mobile elements (plasmids, transposons, integrons; Carattoli, 2009; Cambray et al., 2010; Aminov, 2011). It is well-known that both metals and antibiotic resistance genes are located on the same mobile elements, leading to a co-selection of ARGs by metals (Baker-Austin et al., 2006; Seiler and Berendonk, 2012). Metal contaminations are widely spread in anthropogenic environment contributing on ARGs propagations (Sakan et al., 2009; Ji et al., 2012; Seiler and Berendonk, 2012).

The question of the environmental and human risks of an increasing release of bacteria carrying ARGs into the natural environment has been a subject of intense scientific and political debate in recent years. Consequently, there are a growing number of reports concerning the prevalence and dissemination of ARBs and ARGs into various environmental compartments (e.g., Kümmerer, 2004; Martínez, 2008; CDC, 2013; WHO, 2014; Devarajan et al., 2015a). Effluents from hospitals, industry, municipal organizations, and urban/agricultural runoff in many developing countries represent a significant source of emerging contaminants (metals, ARGs, ARB) in the receiving environment as the effluents are discharged into sewer systems, rivers, lakes, and seas without prior treatment which may then accumulate in sediments (Spindler et al., 2012; Mwanamoki et al., 2014; Devarajan et al., 2015a). Rivers and lakes are considered to be putative reservoirs of emerging contaminants (medicinal products, metals, ARGs) since they collect wastewaters containing various contaminants from various origins (Kümmerer, 2004; Poté et al., 2008; Allen et al., 2010). Furthermore, the sediments may accumulate 100 to 1000 times as many heavy metals, FIB and ARGs as the overlying water (e.g., Poté et al., 2008; Haller et al., 2009; Thevenon et al., 2012a,b; Mubedi et al., 2013; Mwanamoki et al., 2014, 2015; Devarajan et al., 2015a,b) and offer the opportunity for reconstructing the pollution history and evaluating the impacts.

Many studies have been performed to quantify ARB and ARGs in different environmental compartments around the world and explained the role of aquatic ecosystems as reservoirs of antibiotic resistance (e.g., Schwartz et al., 2003; Kümmerer, 2004; Levy and Marshall, 2004; Martínez, 2008; Stoll et al., 2012; Marti et al., 2014; WHO, 2014). Most of these studies formulated recommendations and hypotheses including the suggestion of further researches in different regions according to the source of drinking and recreational water, the practice of wastewater management, economic situation and sociocultural aspects of population, and climatic conditions. Nevertheless, many studies have not considered the influence of tropical conditions (e.g., in developing nations such as Sub-Saharan African countries) on the accumulation of these emerging contaminants in aquatic environment, which can vary considerably with developed countries (under temperate conditions; e.g., Czekalski et al., 2014; Devarajan et al., 2015a). Consequently, little data are available on the assessment of heavy metals and neither there is no much information found regarding quantitative and qualitative aspects of ARB as well as ARGs in the aquatic environment under tropical conditions, which have an average daily peak temperatures reaching 30⋅C. Measures to reduce the potential human and environmental risks caused by hazardous substances (such as toxic metals), ARB, and ARGs include their characterizations and selection of target ARB and ARG, identification of potential sources as well as risk assessment, the development of reliable surveillance and risk assessment procedures, and finally, the implementation of technological solutions that can prevent environmental contamination with ARB and ARGs (WHO, 2014; Berendonk et al., 2015; Devarajan et al., 2015a).

The aim of the research presented in this paper is to assess the role of untreated hospital effluents discharged into freshwater receiving system under tropical conditions. This assessment was based on: (i) sediment physicochemical characterization including sediment grain size, total organic matter (OM; loss on ignition), and toxic metals including Cr, Co, Ni, Cu, Zn, As, Cd, Pb, and Hg - (ii) quantitative polymerase chain reaction (qPCR) on ARGs (*bla*_TEM_, *bla*_CTX-M_, *bla*_SHV_, and *aadA*), total bacterial load, and selected bacterial marker genes of fecal indicator bacteria [FIB; *E. coli* and *Enterococcus* (ENT)] and *Pseudomonas* species (Psd). To the knowledge of the authors this is first report on the accumulation of emerging microbial contaminants in the sediments of freshwater receiving systems in a central African region and specifically in the city of Kinshasa, the capital of the Democratic Republic of the Congo. Nevertheless, it should be noted that, one may argue that studies that analyze the DNA such as this study, while providing information on the presence/absence or even quantitative data but do not provide information on the expression of these ARGs (Lachmayr et al., 2009). However, the expression of genes is not the central query of this study when the purpose of this study is to address the evaluation of the tropical aquatic environment to serve as reservoirs of heavy metals and ARGs (that could be potentially transferred to other bacterial cells through horizontal gene transfer; Devarajan et al., 2016). The parameters analyzed were correlated in order to identify the potential sources of receiving system contamination.

## Materials and methods

### Study site and sampling

**Figure 1:**
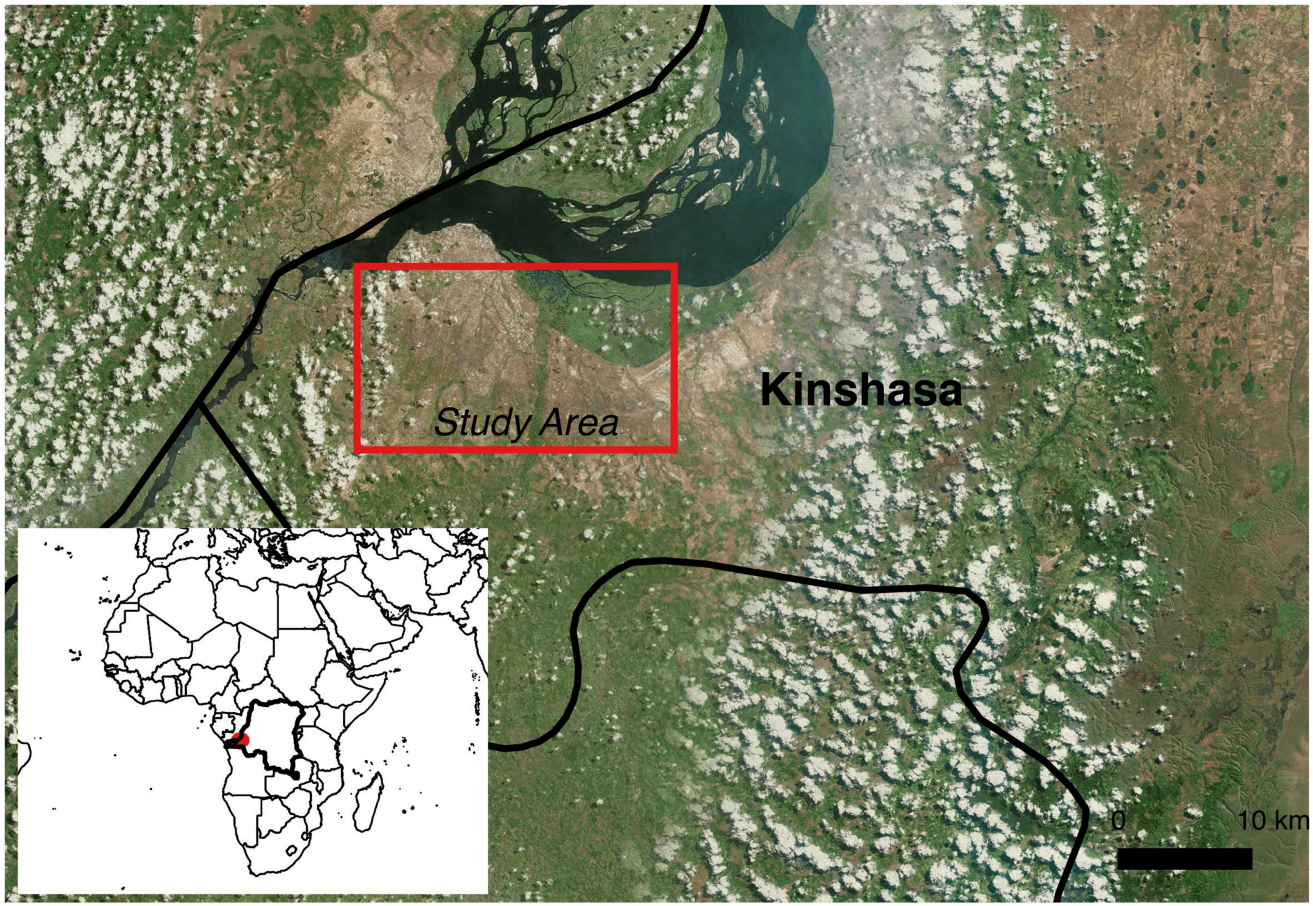
**Localization of the sampling site in the province of Kinshasa, Republic Democratic of Congo (Adapted from Google Maps)**.

Kinshasa, the capital and largest city in the Democratic Republic of the Congo (DRC; Figure 1), is the 27th largest urban area in the world with 11,587,000 inhabitants and covering 9′965 km^2^. The climate is classified as humid and dry with an average temperature of 21–30⋅C. The city has ~20 hospitals and various medical centers and polyclinics with different intrinsic characteristics (size, type of services, medical practices…). The wastewater effluents are discharged from the big hospitals into drainage systems and ejected into the urban river receiving systems without prior treatment. For small hospitals and many medical centers, wastewaters are directly rejected onto the soil, septic tanks, wells, or into rivers, and even using buckets. The selection of hospitals was done on the basis of the hospital size relatively to the practice of wastewater management as described above, and the hospital location in relation with the river receiving system. Three rivers receiving hospital effluent waters were selected for this study. They are affected by one very large hospital and three recent and innovative hospitals. No industry is located near the hospitals selected.

The sampling took place in December 2014. The surface sediments (0–4 cm layer) were collected from (i) outlet pipes (HOP) of the 4 hospitals selected, labeled H1, H2, H3, and H4. The collection points were adjacent to the hospital effluent outlet pipe before discharge into rivers. This sampling point is named E, (ii) at the HOP discharge points into the rivers (point named RP), (iii) in the rivers 50 m upstream from HOP discharge points (point named US), and (iv) in the rivers 50 m downstream from HOP discharge points (point named DS). Approximately 400–500 g of sediment were taken from each site in triplicate. All samples were stored in an icebox at 4⋅C until shipping (Mubedi et al., 2013; Mwanamoki et al., 2015; Devarajan et al., 2015b) and analyzed within 2 weeks.

### Sediment grain size, organic matter, and water content

The sediment particle grain size was measured using a Laser Coulter® LS-100 diffractometer (Beckman Coulter, Fullerton, CA, USA), following 5 min ultrasonic dispersal in deionized water according to the method described by Loizeau et al. (1994). The sediment total organic matter (OM) content was estimated by loss on ignition at 550⋅C for 1 h in a Salvis oven (Salvis AG, Emmenbrücke, Lucerne, Switzerland). Sediment total water content was measured by drying the samples at 60⋅C for overnight and the weight loss was taken for the percentage of water content.

### Toxic metal analysis

Before being analyzed, sediment samples were lyophilized at −45°C after homogenization and air-drying at ambient room temperature. Toxic metals including Cr, Cu, Zn, Cd, and Pb were determined by Inductively Coupled Plasma Mass Spectrometry (ICP-MS, Agilent model 7700 series) following the digestion of sediments in Teflon bombs heated to 150°C in analytical grade 2M HNO_3_ (Loizeau et al., 1994; Pardos et al., 2004; Poté et al., 2008). Multi-element standard solutions at different concentrations (0, 0.02, 1, 5, 20, 100, and 200 μg/L) were used for calibration. Total variation coefficients of triplicate sample measurements were under 5% and chemical blanks for the procedure were less than 2% of the sample signal. The metal concentrations of sediments were expressed in ppm (mg kg^−1^ dry weight sediment).

Total Hg analysis was carried out using the Atomic Absorption Spectrophotometer (AAS) for mercury determination (Advanced Mercury Analyser; AMA 254, Altec s.r.l., Czech Rep.) following the method described by Hall and Pelchat (1997) and Roos-Barraclough et al. (2002). The method is based on sample combustion, gold amalgamation, and AAS. The detection limit (3 SD blank) was 0.005 mg kg^−1^ and the reproducibility better than 2%.

### Total DNA extraction

Total DNA from sediment samples was extracted using the PowerSoil® DNA Isolation Kit (MoBio Laboratories, Carlsbad, USA) according to manufacturer's instructions. DNA extraction was performed with three replicate sample (from the same sediment sample) to compensate for heterogeneity. The concentration of extracted DNA was measured using a Qubit Fluorimeter (Life Technologies Europe B.V., Zug, Switzerland). The isolated DNA was stored at −20°C until used.

### qPCR quantification of selected genes in sediments: 16S rRNA, ARGs, and FIB

Quantification of ARGs (*bla*_TEM_, *bla*_CTX-M_, *bla*_SHV_, and *aadA*), bacterial gene markers for *E. coli, Enterococcus and Pseudomonas* species, and 16S rRNA genes by qPCR was performed [qPCR reactions, control plasmids, calculation for absolute gene copy numbers (gene concentration) and the gene copy numbers normalized to 16S rRNA (abundance)] as previously described by Devarajan et al. (2015a). Briefly; Genes were quantified with Eco qPCR system (Illumina, Switzerland) using KAPA SYBR® FAST qPCR Master Mix Universal Kit (KAPA Biosystems, USA). The primer sequences and reaction conditions are provided in Table 1. The following cycling parameters were applied: 10 min at 95°C for the polymerase activation; followed by 40 cycles of 95°C for 30 s, optimal Tm for 30 s and 72°C for 30 s. The temperature melting curve profile was obtained using the following conditions; 95°C for 30 s, optimal Tm for 30 s, followed by 95°C for 30 s.

**Table 1:** **Primers used in this study**.

**Target organism/gene**	**Primer**	**Oligonucleotide sequence (5′-3′)**	**Tm (°C)**	**Size (bp)**	**References**
Bacterial 16S rRNA	338F	ACTCCTACGGGAGGCAGCAG	55	197	Ovreås et al., 1997
	518R	ATTACCGCGGCTGCTGG			
*E. coli* (*uidA*)	Uida405F	CAACGAACTGAACTGGCAGA	55	121	Chern et al., 2011
	Uida405R	CATTACGCTGCGATGGAT			
ENT (16S rRNA)	Ent376F	GGACGMAAGTCTGACCGA	55	221	Ram et al., 2004
	Ent578R	TTAAGAAACCGCCTGCGC			
*Pseudomonas* spp.	Pse435F	ACTTTAAGTTGGGAGGAAGGG	55	251	Bergmark et al., 2012
	Pse435R	ACACAGGAAATTCCACCACCC			
*bla*_TEM_	TEM-RT-F	GCKGCCAACTTACTTCTGACAACG	55	247	Sidrach Cardona et al., 2014
	TEM-RT-R	CTTTATCCGCCTCCATCCAGTCTA			
*bla*_CTX-M_	blaCTX-M-rt-f	ATTCCRGGCGAYCCGCGTGATACC	62	227	Fujita et al., 2011
	blaCTX-M-rt-r	ACCGCGATATCGTTGGTGGTGCCAT			
*bla*_SHV_	blaSHV-rt-f	CGCTTTCCCATGATGAGCACCTTT	60	110	Xi et al., 2009
	blaSHV-rt-r	TCCTGCTGGCGATAGTGGATCTTT			
*aadA*	aadA-F	GCAGCGCAATGACATTCTTG	55	282	Madsen et al., 2000
	aadA-R	ATCCTTCGGCGCGATTTTG			

All the reactions included negative (with no template DNA) and positive controls (10-fold serial dilutions of pGEM-T plasmid with respective target gene insert). All negative controls resulted either in no amplification or a threshold cycle (Ct) higher than the most diluted standard (pGEM-T plasmid). A sample was considered to be below the limit of detection (LOD) or negative for a target gene if ≥2 out of 3 technical replicates were negative or if sample Ct values were ≥Ct of negative controls. Samples above LOD were considered to be below the limit of quantification when the standard deviation of Ct values of methodological triplicates was 40.5 and their Ct value was higher than the Ct of the most diluted standard whose standard deviation of Ct values was ≤ 0.5. For each reaction the efficiency of the assay was measured using the slope of the standard curve measures (E = 10^[−1∕slope]^ − 1). The absolute copy number of each reaction was quantified by referring to the corresponding standard curve obtained by plotting the copy number of the constructed pGEM-T plasmid vs. threshold cycles. The serial 10-fold dilutions of plasmid DNA containing the respective target gene copies were used for the standard curve. To emphasize the relative abundance of the resistance genes the concentrations of the gene copy numbers were presented as percentage of “copy number of a gene/copy number of 16S rRNA” for each sample.

### Data analysis

The 16S rRNA (total bacterial load), FIB and the selected marker genes and ARGs in the samples are expressed as “gene copy numbers” in per gram of dry sediment weight normalized to the DNA extraction yield. The “relative abundance” of the selected genetic marker genes (normalized to 16S rRNA) were emphasized by the ratio = (copy number of a gene) / (copy number of 16S rRNA) for each sample (Czekalski et al., 2014; Devarajan et al., 2015a). A statistical treatment of data; Correlation matrix (Pearson), Principal component analysis (PCA) and its extensions to between (BGA), and within groups (WGA) analyses (ade4 package in R) was used to analyze the grouping hospital/sampling point samples by monitoring quality variables (metal content, FIB abundance, ARGs, mean grain size and OM content). The statistical software was R version 3.2.2 (Team, 2015). Linear fixed model were fitted to the data using the functionality of the package lme4 (Bates et al., 2015). Average concentrations of gene copy numbers was modeled using the hospital and the sampling point as fixed effects and technical and biological effects as random effects. Significance of fixed effects was assessed by a *t*-test using a significance level of 5%. Model checking was based residual plots and normal probability checking using the raw residuals. Models were reduced using the likelihood ratio test. Pairwise comparison were evaluated based on adjusted *p*-values obtained using single-step method (Hothorn et al., 2008).

## Results and discussion

### Sediment physicochemical parameters and metal content

Sediment characteristics including particle grain-size and total organic matter (OM) are presented in Table 2. The sediment grain size and the OM varied substantially internally within the sampling sites (*p* < 0.05). Surface sediments of rivers in all sites studied are generally sandy-silt. The maximum value of clay observed at sites H1, H2, and H3 was less than 3%. H4 presented the maximum value of clay (11%). The same distribution was observed for sediment OM content (*p* < 0.05). The values ranged from 15.0 to 46.2% (H1), 4.7–9.4% (H2), and 0.7–8.7%. No great difference in OM was observed in the sampling sites of H4 (8.3–8.5%). Previous studies have reported that there are large variations in the distribution of sediment OM and grain size in freshwater, lakes, rivers, and reservoirs. The OM in non-contaminated freshwater sediments varies from 0.1 to 6.0% (Poté et al., 2008; Haller et al., 2009; Mubedi et al., 2013). The sediment from all sites (US: upstream; E: exit (outlet); RP: reject point (outlet discharge) and DS: downstream) of H1 and H4 are contaminated by organic matter. These results support the hypothesis that hospital effluents are one of many sources of contamination and that the contamination occurs at multiple points of entry along the river bank.

**Table 2:** **Physico-chemical parameters of surface sediments from sampling points of each hospital site**.

**Hospital**	**Sampling point^b^**	**Clay (%)**	**Silt (%)**	**Sand (%)**	**Mean grain size (μm)**	**OM^c^ (%)**
Hospital 1	US	3.21	27.46	69.33	46.57	15.03
	E	2.61	47.96	49.43	65.69	46.15
	RP	3.36	58.44	38.20	42.70	34.78
	DS	1.31	25.45	73.24	139.10	18.10
Hospital 2	US	0.41	14.00	85.59	157.20	6.05
	E	ND^a^	ND	ND	ND	ND
	RP	0.48	13.46	86.06	229.10	9.37
	DS	1.85	63.27	34.88	48.67	4.74
Hospital 3	US	0.13	10.66	89.21	226.00	0.75
	E	0.78	9.46	89.76	205.80	3.91
	RP	0.38	7.31	92.31	234.20	1.79
	DS	0.27	27.47	72.26	135.20	8.72
Hospital 4	US	5.68	38.71	55.61	52.67	8.33
	E	0.37	58.23	41.40	58.71	8.40
	RP	10.83	57.51	31.66	24.04	8.44
	DS	0.94	26.00	73.06	82.54	8.51

a ND: not determinated.

b US, upstream; E, exit; RP, reject point; DS, downstream.

c OM, organic matter.

The results of the toxic metal analysis are reported in Table 3. The maximum concentration was observed in sediments at H1, reaching values (in mg kg^−1^) of 47.9 (Cr), 213.6 (Cu), 1434.4 (Zn), 2.6 (Cd), 274.2 (Pb), and 13.6 (Hg). Sewages and rivers, which receive the majority of drained urban wastewaters of the city of Kinshasa, are used by the local population as uncontrolled landfills for domestic solid wastes. This can explain the presence of high metal concentrations in sediment (Mavakala et al., 2016). As the studied rivers flow through the City of Kinshasa, additional pollutant sources such as domestic sewages, uncontrolled landfills and artisanal activities located in the banks of rivers can probably explain the presence of contaminants accumulation in sediment. However, the presence of other non-identified sources and untreated hospital effluent water discharge cannot be excluded. In general, the concentration of toxic metals at points E, RP, and DS are higher for all sampling sites than the upstream sampling points indicating the effect of hospital effluent waters on the contamination of rivers by toxic metals.

**Table 3:** **Metal content of surface sediment samples from sampling point of each hospital site, analyzed by ICP-MS (expressed in mg. kg^−1^)**.

**Hospital**	**Sampling point**	**Cr**	**Co**	**Ni**	**Cu**	**Zn**	**Cd**	**Pb**	**Hg**
Hospital 1	US	34.32	4.29	14.24	**107.45**	**884.61**	**1.79**	**189.62**	**1.43**
	E	**45.54**	4.55	17.42	**213.59**	**1004.47**	**1.88**	**137.08**	**13.60**
	RP	**47.87**	4.97	17.42	**204.13**	**1077.17**	**2.07**	**124.40**	**3.94**
	DS	**47.58**	7.11	21.20	**184.67**	**1434.78**	**2.65**	**274.19**	**3.25**
Hospital 2	US	13.03	1.79	6.29	**59.18**	**410.69**	0.57	**86.67**	**0.64**
	E	ND	ND	ND	ND	ND	ND	ND	ND
	RP	11.96	1.81	13.48	34.18	**304.92**	0.54	**281.52**	**0.44**
	DS	10.43	1.61	5.67	**51.91**	**352.41**	0.46	**81.15**	**0.74**
Hospital 3	US	3.20	0.37	16.73	**46.06**	87.21	0.18	**36.38**	0.15
	E	7.81	0.83	14.63	22.08	**147.29**	0.29	34.48	**0.68**
	RP	4.18	0.44	13.57	8.86	68.27	0.08	15.82	**0.31**
	DS	4.97	0.62	3.79	22.65	**153.77**	0.22	**40.75**	**0.37**
Hospital 4	US	15.36	0.99	4.85	9.38	71.99	0.10	14.96	**0.39**
	E	27.92	2.68	9.98	**47.54**	**365.75**	0.52	**75.98**	**0.56**
	RP	21.91	1.20	5.96	12.66	98.75	0.16	20.98	**0.51**
	DS	15.23	1.13	5.10	12.86	100.30	0.14	20.73	**0.44**
	SQGs^a^	37.30			35.70	123.00	0.60	35.00	0.17
	PEL^b^	90.00			197.00	315.00	3.50	91.30	0.49

a Sediment quality guidelines (mg.kg^−1^).

b Probable effect level (mg.kg^−1^).

In bold: values above SQGs.

Cr, chromium; Co, cobalt; Ni, nickel; Cu, copper; Zn, zinc; Cd, cadmium; Pb, lead; Hg, mercury.

Metals accumulation in sediments is one of a good indicators for predicting the deterioration of a contaminated environment with inorganic pollutants. The release of heavy metals into the aquatic ecosystem can lead to the pollution of water resources and may place aquatic organisms and human health at risk. The main human and environmental risk is remobilization of the contaminants and their return to the hydrosphere either by sediment re-suspension or by infiltration into groundwater (Wildi et al., 2004; Poté et al., 2008).

The evaluation of the potential deleterious effects of the metals toward benthic fauna, which is based on sediment quality guidelines (SQG; CCME, 1999; MacDonald et al., 2000; Long et al., 2006) provides an estimate of the hazard that the sediments may represent to the local biota. The authors proposed a “Threshold effect concentration” (TEC) for specific metals which is the level above which an organism is affected (or responds) and below which it does not, and a “probable effect level” (PEL), a contaminant level that is likely to have an adverse effect on biota (Mwanamoki et al., 2014). According to the SQGs and PEL (Table 3), the concentration of Cr, Cu, Zn, Pb, and Hg in sediment may present a potential toxic effect on indigenous fauna and flora living in these aquatic environments. Furthermore, the high PEL values observed upstream of hospital effluent discharges indicate that hospital effluents are only one of several sources of contamination. Indeed, many anthropogenic activities (i.e., industrial, sewage, agricultural land field, mining…) are known to be responsible of heavy metal release in the environment (Poté et al., 2008; Lim et al., 2013; Devarajan et al., 2015b; Niane et al., 2015) and may be responsible of the high metal values observed upstream HOP effluents discharge.

### Abundance of bacterial population

**Figure 2:**
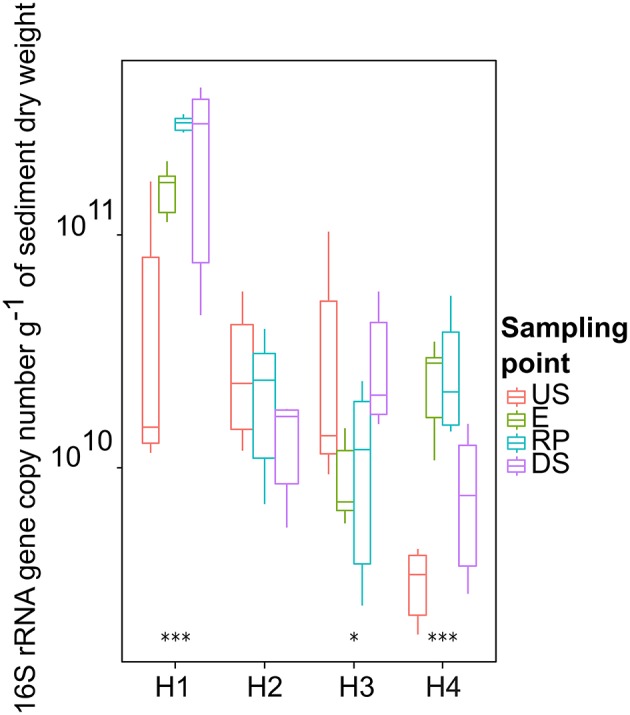
**Raw 16S rRNA copy number detected in hospital receiving systems (16S rRNA gene copy number / g of DS) at each sampling point**. Significant stars represent significant variation between US and RP point, ***-*p* < 0.01; and * 0.1> *p* >0.05. For the pairs not marked the statistical difference between US and RP was statistically insignificant. The line in each box marks the media and boxes: 25th and 75th percentiles; whiskers: 5th and 95th percentiles and outliers ± 1.5*IQ. US, upstream; E, hospital outlet effluent; RP, reject point; DS, downstream.

The total bacterial load in sediment samples is presented in Figure 2 (based on 16S rRNA gene copy numbers). In general, 16S rRNA gene copy numbers varied significantly between sampling sites (*p* < 0.05) with values of (log copy numbers g^−1^ of dry sediment). The 16S rRNA gene copy numbers in samples from H1 were several orders of magnitude higher than those observed in others hospitals. However, these results are in conformity with those found in sediment from contaminated sediments receiving WWTP effluent waters (Devarajan et al., 2015a). Furthermore, the total bacterial load after the effluent discharge (RP) for H1 and H4 were respectively 10 and 7.85 times higher than the values observed in their respective control sites (US). In contrast, the total bacterial load in the points RP for H2 and H3 was 3.7 to 6.8 times lower than their respective control site (US), indicating the possible presence of other sources of contamination.

**Figure 3:**
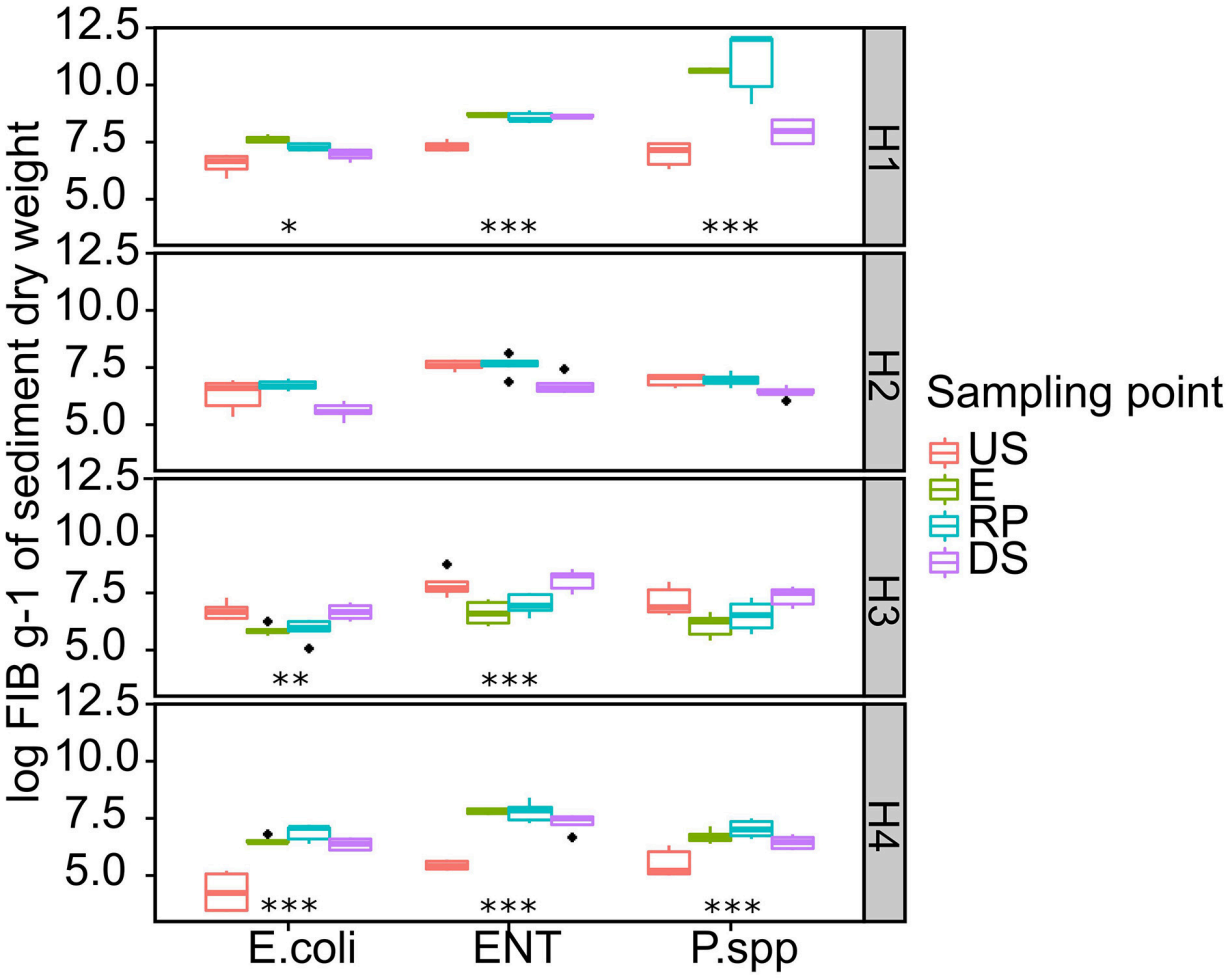
**Raw FIB copy number detected in hospital receiving systems at each sampling point**. Significant stars represent significant variation between US and RP point, ***-*p* < 0.01, ** 0.01> *p* >0.01 and * 0.1> *p* >0.05. For the pairs not marked the statistical difference between US and RP was statistically insignificant. The line in each box marks the media and boxes: 25th and 75th percentiles; whiskers: 5th and 95th percentiles and outliers ± 1.5*IQR. US, upstream; E, hospital outlet effluent; RP, reject point; DS, downstream; ENT, Enterococcus; Psd, Pseudomonas species.

The average copy number of FIB bacterial marker genes including *E. coli*, ENT, and Psd in the sediment samples is presented in Figure 3. The bacterial density varies considerably depending on the sampling point and the type of hospital. For example, the *E. coli* range at the US site (log copy numbers g^−1^ of dry sediment) was 6.44, 6.30, 6.67, and 4.21 for H1, H2, H3, and H4, respectively; ENT range (copy numbers g^−1^ of dry sediment) of 7.28, 7.59, 7.87, and 5.41 for H1, H2, H3, and H4, respectively; and Psd range (log copy numbers g^−1^ of dry sediment) was 6.96, 6.94, 7.10, and 5.49 for H1, H2, H3, and H4, respectively. The input of hospital wastewater in the receiving system lead to a 6, 1, and 288 times greater abundance of *E. coli* for H1, H2, and H4, respectively. In H3 RP sampling point, a 6.2 times decrease in *E. coli* abundance was measured after wastewater discharge showing that H3 wastewater did not contribute more than the environmental enrichment in *E. coli.*

**Figure 4:**
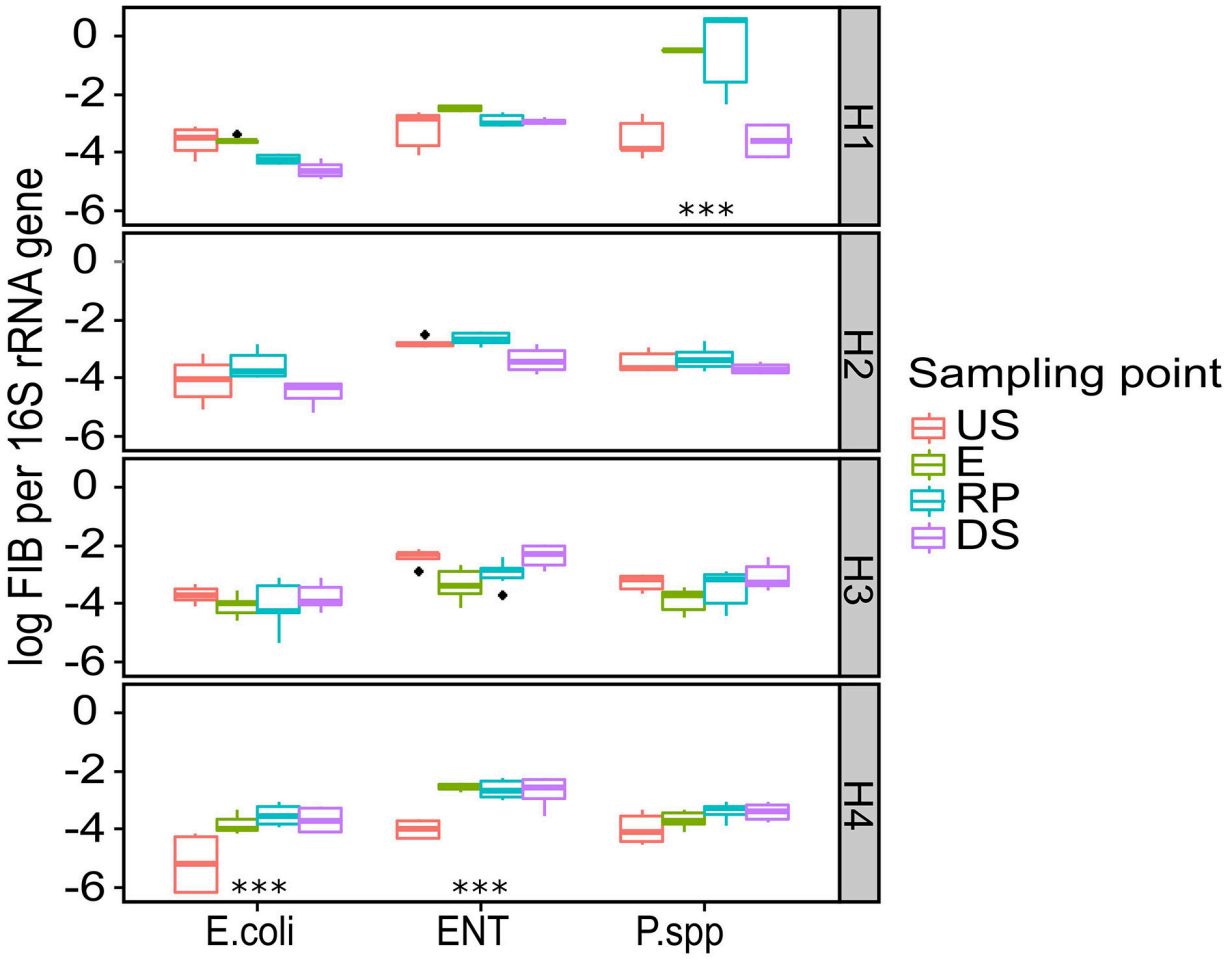
**Normalized FIB copy number detected in hospital receiving systems at each sampling point**. Significant stars represent significant variation between US and RP point, ***-*p* < 0.01. For the pairs not marked the statistical difference between US and RP was statistically insignificant. The line in each box marks the media and boxes: 25th and 75th percentiles; whiskers: 5th and 95th percentiles and outliers ± 1.5*IQR. US, upstream; E, hospital outlet effluent; RP, reject point; DS, downstream; ENT, Enterococcus; Psd, Pseudomonas species.

Raw load of selected bacterial marker genes followed the 16S rRNA trend. To avoid inconstancies between qPCR assays, including suboptimal efficiencies, selected bacterial species marker genes and ARGs were normalized using 16S rRNA (Figures 4, 6). The greatest abundance of bacterial populations was recorded at H1 and H4 sampling sites (except *E. coli* for H1 and Psd for H4). H2 and H3 did not show any significant increase in bacterial marker genes abundance, which was also the case for 16S rRNA abundance (0.20 < *p* < 0.98 for H3 and 0.06 < *p* < 0.91 for H3). The abundance of bacterial marker genes can be either accumulated or diluted depending on the river characteristics (depth, flow rate, turbulences). No uncultivable quantitative data is available for RDC aquatic systems but the high level of total bacteria and FIB in US sampling points is consistent with data from highly contaminated sediments (Devarajan et al., 2015a,b). Compared to other hospitals, the H1 sampling site had higher values for total bacterial load which could be explained by the larger size of the hospital and the shared drainage of urban and hospital wastewater. The three other hospitals are smaller than H1 which results in smaller amounts of contaminants in their effluents. In the case of H2, no canalization of hospital wastewater was used. The cumulative effect of no drainage system and the presence of wild discharge next to the US sampling point may explain why hospital effluents discharge showed no significant increase in FIB abundance in the receiving system (Orsi et al., 2007; Graham and Polizzotto, 2013). This effluent didn't contribute more to already environmental abundance and the observed decrease in FIB abundance may be due to contaminant dilution and transport downstream by river flow (Knapp et al., 2012; Chen et al., 2013).

FIB including *E. coli* and ENT are commonly used to assess microbial safety of aquatic systems. It is well-known that many *E. coli* and ENT are also responsible for numerous health care-associated infections of the bloodstream, urinary tract, and surgical incision sites (Alm et al., 2014). To develop this high level of pathogenicity, these bacteria have acquired islands of pathogenicity including antibiotic, heavy metal resistance genes, and virulence factors. Several studies in other parts of the world have revealed the presence of pathogenic micro-organisms which are multi-resistant to antibiotics in hospital effluents (Emmanuel et al., 2009). In the absence of wastewater treatment as is the case in the hospitals in the study presented in this paper, these bacteria will be discharged directly into aquatic receiving systems. It is well-known that FIB are able to survive and proliferate in sediments as sediments provide favorable conditions for proliferation and growth. (Poté et al., 2009). Furthermore, FIB have a great ability to acquire ARG (Levy and Marshall, 2004) and are able to transfer their resistance to autochthonous bacteria by HGT (Sidrach Cardona et al., 2014). Consequently, the discharge of raw hospital wastewater could lead to an environmental reservoir of clinical resistant bacteria and their associated genes developing in the sediment (Marti et al., 2014).

### Quantification of antibiotic resistance genes

**Figure 5:**
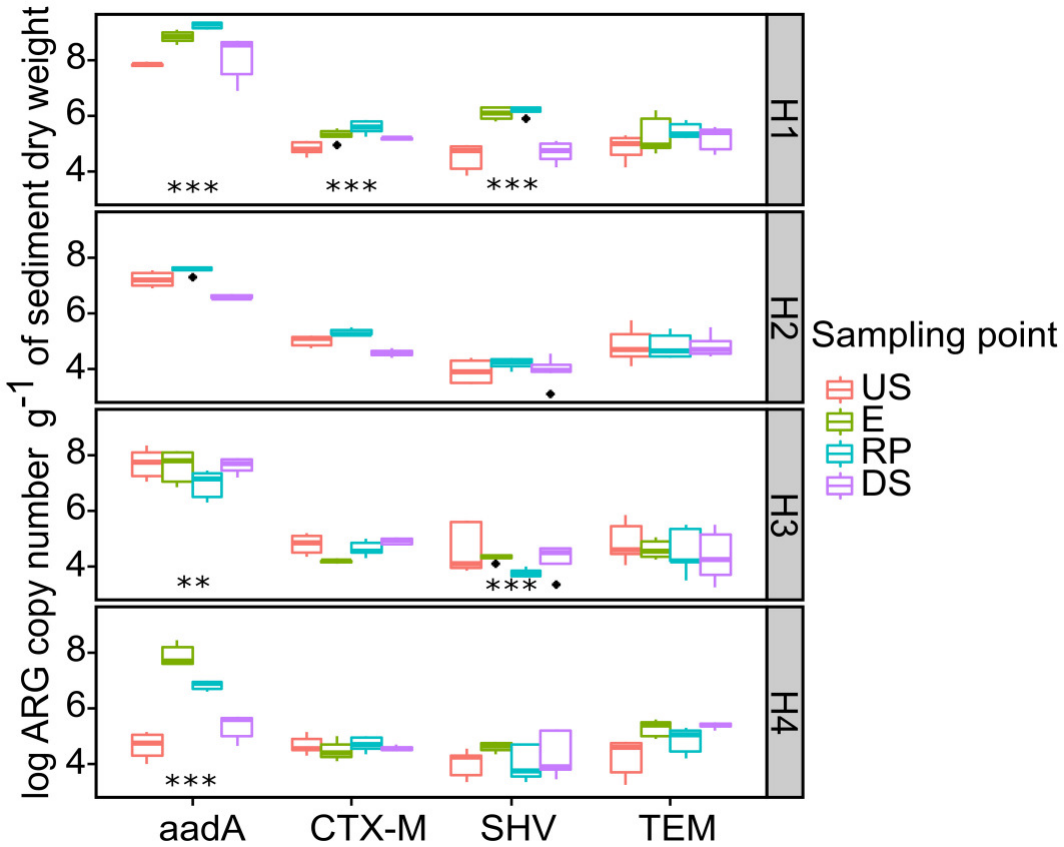
**Raw ARGs copy number detected in hospital receiving systems at each sampling point**. Significant stars represent significant variation between US and RP point, ***-*p* < 0.01; and ** 0.01> *p* >0.01. For the pairs not marked the statistical difference between US and RP was statistically insignificant. The line in each box marks the media and boxes: 25th and 75th percentiles; whiskers: 5th and 95th percentiles and outliers ± 1.5*IQR. US, upstream; E, hospital outlet effluent; RP, reject point; DS, downstream.

qPCR was performed to quantify the selected ARGs conferring resistance to ß-lactam (*bla*_TEM_, *bla*_CTX-M_, and *bla*_SHV_) and aminoglycoside (*aadA*) in DNA extracted from the sediment samples. ARG selection was based on various criteria including (Devarajan et al., 2016): (i) clinically relevant genes (human risk); (ii) genes conferring resistance to frequently used antibiotics; (iii) ARGs previously reported in mobile genetic elements; and (iv) the antibiotics used in 6 pilot hospitals in Kinshasa (Nzolo et al., 2013). The raw gene copy number (ARGs g^−1^ of dry sediment) was used to estimate the general changes in ARGs level in receiving systems. The results are presented in Figure 5. The ARGs copy number (ARGs g^−1^ of dry sediment) for *aadA, bla*_CTX−M_,*bla*_SHV_, and *bla*_TEM_ varied, respectively from 4.64 to 7.83, 4.67 to 5.01, 3.92 to 4.66, and 4.23 to 4.86 at US sites, and from 5.33 to 9.24, 4.55 to 5.61, 3.76 to 6.17, and 4.36 to 5.46 at RP/DS sampling sites. The great abundance of *bla*_TEM_ and *aadA* genes at all sampling sites (US, E, RP, and DS) could be explained by their ubiquitous presence as housekeeping genes, which has previously been shown to occur frequently among soil bacteria as well as by their presence in sewage and effluent receiving systems (Demanèche et al., 2008; Thevenon et al., 2012a; Suzuki et al., 2015). A relevant increase in ARG level after wastewater discharge is only observed in H1 sediments (*p* < 0.05) with a 25.6, 45.0, and 148 times increase observed between US and RP sampling points for *bla*_CTX-M_,*bla*_SHV_, and *aadA*, respectively. For the others hospitals, the general trend of ARGs increased after wastewater discharge was no longer observed. The abundance of ARGs and associated bacteria in a receiving system may vary depending on the size of the hospital and type of services.

**Figure 6:**
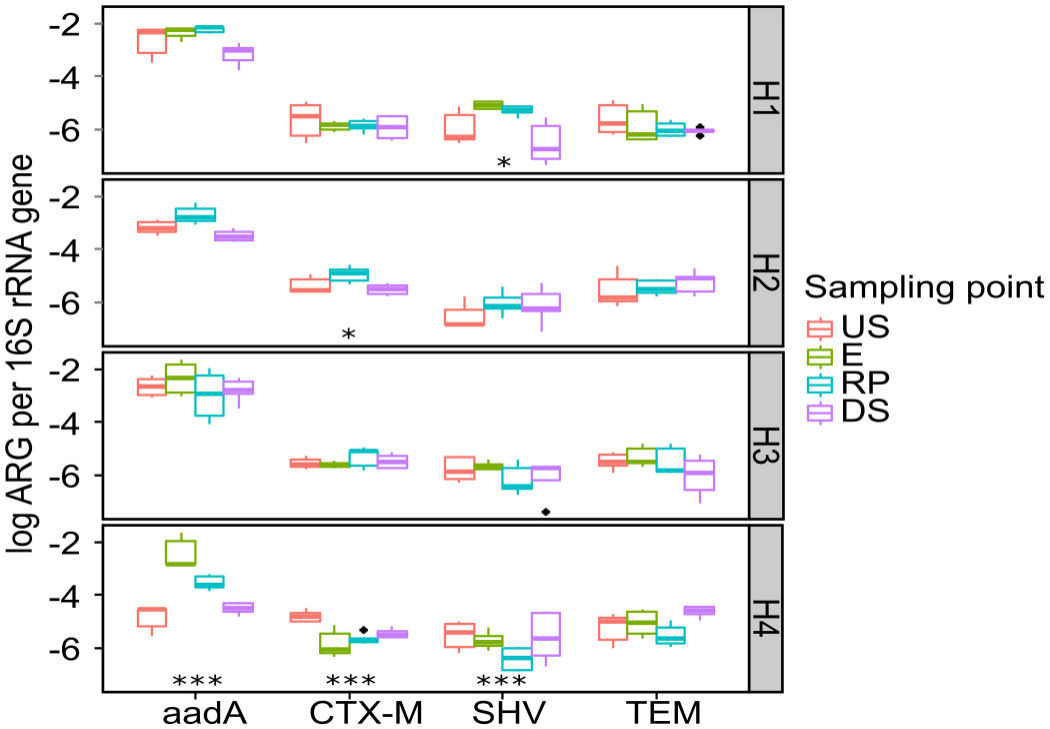
**Normalized ARGs copy number detected in hospital receiving systems at each sampling point**. Significant stars represent significant variation between US and RP point, ***-*p* < 0.01; and * 0.1> *p* >0.05. For the pairs not marked the statistical difference between US and RP was statistically insignificant. The line in each box marks the media and boxes: 25th and 75th percentiles; whiskers: 5th and 95th percentiles and outliers ± 1.5*IQR. US, upstream; E, hospital outlet effluent; RP, reject point; DS, downstream.

The normalized/relative abundances of 16S rRNA were found in order to quantify relative change in ARGs abundances, that is, whether or not more or fewer ARGs appear per microbial genome (Laht et al., 2014). The relative abundance of ARGs in the sediment samples is presented in Figure 6. In general, the influence of hospital effluents was not observed in the relative abundance of ARGs copy numbers in receiving systems. Some specific cases such as *aadA* in H4, *bla*_SHV_ in H1, and *bla*_CTX-M_ in H2 increased significantly after hospital effluent discharge (*p* < 0.01). These data suggest that increases in ARGs abundances are directly related to wastewater discharge depending on hospital type and disposal practices, although unknown sources and/or causes also exist (Graham et al., 2011; Marti et al., 2014). The prevalence of microbial contaminants in the control sites (upstream) could be explained by, for example, input from major activities such as agricultural runoff, open defecation, urban discharge, and other anthropogenic activities along the river banks, which receives considerable amounts of wastewaters (Dekov et al., 1998; Tshibanda et al., 2014). The abundance of total bacterial load, ARGs, and FIB showed a relevant increase after wastewater discharge but the results highlight that hospital wastewater effluents are not the only source of micropollutant accumulation.

Interestingly, the relative abundances of ARGs observed in this study were greater than in other studies performed in a similar environment under tropical conditions (Graham et al., 2011; Devarajan et al., 2016) and can be compared to data obtained in industrialized countries (Devarajan et al., 2015a). However, the lack of background information and the knowledge gap in our pristine study site does not help in understanding the trends observed. ESBLs are mostly TEM, SHV, and CTX-M derivatives. The great abundance of clinically relevant ARGs such as *bla*_CTX-M_ and *bla*_SHV_ in the sediments studied may indicate the possible presence of ESBL in these systems (Poirel et al., 2012). The presence of ESBL in rivers (such as the sites studied) is highly alarming because the majority of ESBL are resistant to first line antibiotics but are also resistant to a large number of relevant antibiotics (Tacao et al., 2014). Furthermore, such a large abundance of ARGs represents a serious threat from resistance propagation because gene exchange can take place in sediment between both dead and living bacteria (Mao et al., 2014). It has been determined that approximately 90 bacterial species have natural transformability competences. Among them are many human pathogens, including the genera *Campylobacter, Haemophilus, Helicobacter, Nesseiria, Pseudomomas, Staphylococcus*, and *Streptococcus* (Mao et al., 2014). So the reservoir of ARGs in river sediments can be easily used by other bacteria to become ever more resistant.

The abundance of ARGs in the receiving systems reported in this study can be considered as alarming. Results indicate clearly that sediment receiving system under tropical condition can act as reservoirs of ARGs including *bla*_TEM_, *bla*_CTX-M_, *bla*_SHV_, and *aadA*. It has been demonstrated that the intensive use of antibiotics for humans, animals, and agricultural purpose has led to the release (e.g., through the disposal of human and animal wastes) of ARB and ARGs into soil and aquatic environment (Martínez, 2008; Sommer et al., 2009). In the study region as well as in many Sub-Saharan African countries, there is no regulation for the use of antibiotics in humans, animals as well as for agricultural purpose, and data concerning the occurrence of ARGs and ARBs in aquatic environments is limited (WHO, 2014; Devarajan et al., 2016). Additionally, there is no policies and management tools to facilitate the urban wastewater treatment in study region. Thus, according to the results of this study, we strongly recommend the prudence and regulation for the use of antibiotics in humans and animals consumption, to limit spread of ARGs and ARB into the environment. Furthermore, the need of a strategy for hospital and urban wastewater treatment.

### Statistical correlation

Correlation analysis between total bacterial load, FIB, ARGs, toxic metals, total organic matter, and sediment grain size was carried out to determine potential links between both parameters analyzed and possible origins of contaminants with the results being presented in Table 4. Total organic matter content, metal concentrations, bacterial indicator genetic markers (except *E. coli*), and ARGs were mostly significantly, positively, and mutually correlated. Nevertheless, all ARGs studied at hospital and sampling points are significantly correlated with total bacterial load (16S rRNA): 0.51 to 0.72 (*p* < 0.001, *n* = 65). ARGs (except for *bla*_TEM_) have a positive correlation with *E. coli*, ENT, and Psd (0.57 < *r* < 0.82, *p* < 0.05, *n* = 65) and the metals (Cd, Cr, Cu, Hg, and Zn): (0.37 < *r* < 0.71, *p* < 0.001, *n* = 65). Strong positive and mutual correlation was observed between 16S rRNA, *E. coli*, ENT, and Psd with ARGs (except *bla*_TEM_). These results indicate that these biological contaminants could originate from common sources and they are carried to the receiving system by common transporters (Thevenon et al., 2012b; Devarajan et al., 2015a). In addition, there was a positive correlation between total organic content and the metals in sediments. This observation is also supported by the fact that the contaminants are attached to both large organic and small inorganic particles such as clay and they could behave in a similar way in transporting contaminants to the receiving system (Poté et al., 2008; Zhao et al., 2015).

**Table 4:** **Spearman's Rank-Order Correlation of selected parameters analyzed in sediment samples [*n* = 65, statistically significant coefficients (*p* < 0.05) are in bold]**.

	**Cd**	**Cr**	**Cu**	**Grain size**	**Hg**	**16S rRNA**	**aadA**	**CTX-M**	***E. coli***	**ENT**	**Psd**	**SHV**	**TEM**	**OM**	**Pb**	**Zn**
Cd		**0.90**	**0.95**	−**0.24**	**0.64**	**0.59**	**0.66**	**0.54**	−0.10	**0.47**	**0.64**	**0.60**	**0.25**	**0.76**	**0.63**	**0.99**
Cr			**0.89**	−**0.52**	**0.66**	**0.61**	**0.59**	**0.44**	−0.05	**0.51**	**0.67**	**0.67**	**0.35**	**0.83**	**0.43**	**0.89**
Cu				−**0.29**	**0.78**	**0.67**	**0.71**	**0.57**	−0.11	**0.54**	**0.78**	**0.72**	**0.25**	**0.87**	**0.49**	**0.95**
Grain size					−0.26	−0.33	−0.01	0.03	−0.05	−0.20	−**0.29**	−**0.32**	−**0.33**	−**0.41**	0.23	−**0.26**
Hg						**0.51**	**0.52**	**0.37**	−0.05	**0.46**	**0.70**	**0.63**	0.08	**0.89**	**0.25**	**0.63**
16S							**0.72**	**0.67**	−0.19	**0.83**	**0.80**	**0.63**	**0.51**	**0.72**	0.24	**0.57**
aadA								**0.58**	−**0.36**	**0.74**	**0.73**	**0.66**	0.18	**0.63**	**0.43**	**0.65**
CTX-M									−0.11	**0.57**	**0.62**	**0.56**	**0.24**	**0.59**	**0.52**	**0.51**
*E. coli*										−**0.34**	−0.17	−0.13	−0.04	−0.04	−0.12	−0.11
ENT											**0.73**	**0.59**	**0.47**	**0.59**	**0.27**	**0.45**
Psd												**0.82**	**0.26**	**0.86**	0.21	**0.61**
SHV													0.15	**0.79**	0.19	**0.57**
TEM														0.23	0.12	**0.25**
OM															**0.32**	**0.74**
Pb																**0.63**
Zn																

**Figure 7:**
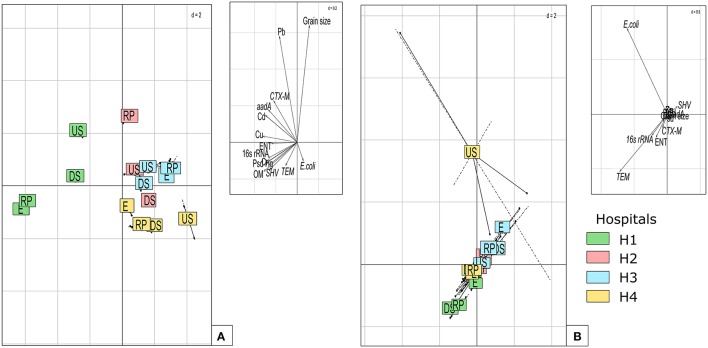
**Grouping of each sampling point according to ARGs, FIB, toxic metals, grain size, and OM. (A)** plot using between group analyses to discriminate each points in various hospital receiving system. **(B)** same samples plotted after decomposing differences in each sampling point by within group analysis. Right upper panels show correlation with variables (PCA variable scores).

BGA on PCA analysis (Figure 7) showed that sampling points varied greatly between hospital sites revealing the impact of (i) initial background level in ARGs, heavy metals, and FIB and (ii) the hospital type (Figure 7-left). To analyze effluent effect independently of the hospital, sampling points were decomposed before fitting (WGA on PCA using hospital as “within group” factor) (Figure 7-right). The results show a large increase in ARGs, FIB, toxic metals, and OM for hospital H1 and H4 linked to wastewater discharge. Lastly, these results indicate that river receiving systems could depend on hospital practices but also that river sediments are already significantly contaminated by unknown sources. As the rivers flow through the City of Kinshasa, additional pollutant sources such as domestic sewage, uncontrolled landfill and artisanal activities located in the banks of rivers can probably explain the presence of contaminants accumulation in sediment (Mubedi et al., 2013; Ngelinkoto et al., 2014; Mwanamoki et al., 2015).

## Conclusion

The research presented in this paper investigates the abundance and dissemination of metal, FIB and ARGs released from hospital effluents into the urban river receiving systems. It's important to note that one of the main concerns of this research consists on the evaluation of the degree to which river receiving system under tropical conditions can act as reservoir of metals, FIB and ARGs. Results demonstrate accumulation of toxic metals, *E. coli, Enterococcus*, and *Pseudomonas* species as well as ARGs in sediment, indicating that river receiving systems under tropical conditions (developing countries such as our study region) can act as a reservoir for metals and emerging microbial contaminants such as FIB and ARGs which can be transferred to human pathogens. Thus, the river receiving systems under tropical conditions which has average daily peak temperatures reaching 30°C, could potentially favor the transfer of mobile genetic elements carrying ARGs to susceptible bacterial pathogens. On the other hand, the presence of higher values of FIB and ARGs in sediment samples located upstream of the hospital outlet discharges (control sites) indicates that the hospital effluent wastewaters are not the only source of deterioration of bacteriological quality of studied rivers. The pollution in the cases studied in this paper may be explained by probable multiple diffuse pollution sources including open defecation, uncontrolled landfills, unregulated effluent discharges, and inadequate sewage collection near the sites studied.

Rivers in most developing nations (such as our study region) serve as a basic network for human and animal consumption as well as irrigation for fresh urban produces. High values of metals, FIB and ARGs observed in river receiving systems indicate the human and environmental potential risks. However, further studies are required to find the pathways used by ARGs to spread, exploring their potential transfer into clinically relevant bacteria and human commensal as well as assessing the human exposure and environment potential risks. To our knowledge, this is the first study to be performed in the region regarding the quantification of ARGs in receiving systems. The quantification of FIB and ARGs as performed in this study can therefore facilitate improved risk assessments for the prudent use of antibiotics in human, animal and agriculture, and provide baseline information for developing strategies (such as hospital and urban wastewater treatment) to limit the spread of these emerging contaminants under tropical conditions.

## Ethics statement

We confirm that the field studies and sampling did not involve misunderstanding. The funder had no role in study design, data collection and analysis, decision to publish, or preparation of the manuscript.

## Author contributions

AL, CM, GG, VS, and JP conceived and designed research; AL, ND, PK, JK, and CM performed research: sampling and laboratory analysis; AL, GG, ND, and JP analyzed data; and AL, CM, VS, and JP wrote the paper. All authors have read, reviewed and approved the manuscript before submission.

## Funding

This research was supported by the Swiss National Science Foundation (grant no. 31003A_150163/1). The funders had no role in study design, data collection and analysis, decision to publish, or preparation of the manuscript. This study represents the tripartite collaboration between University of Geneva (Institute F.A. Forel), University of Kinshasa and Pedagogic National University of Congo (Democratic Republic of Congo).

### Conflict of interest statement

The authors declare that the research was conducted in the absence of any commercial or financial relationships that could be construed as a potential conflict of interest.
